# Dying in honour: experiences of end-of-life palliative care during the 2013–2016 Ebola outbreak in Guinea

**DOI:** 10.1186/s41018-021-00099-3

**Published:** 2021-05-08

**Authors:** Elysée Nouvet, Kevin Bezanson, Matthew Hunt, Sekou Kouyaté, Lisa Schwartz, Fatoumata Binta Diallo, Sonya de Laat, Oumou Younoussa Bah-Sow, Alpha Ahmadou Diallo, Pathé Diallo

**Affiliations:** 1 School of Health Studies, Western University, London, Canada; 2 Thunder Bay Regional Health Sciences Centre, Thunder Bay, Canada; 3 School of Physical and Occupational Therapy, McGill University, Montreal, Canada; 4 Laboratoire Socio-Anthropologique de la Guinée, Conakry, Guinea; 5 Health Research Methods, Evidence and Impact, McMaster University, Hamilton, Canada; 6 Faculté des Sciences et Techniques en Santé, Université Gamal Abdel Nasser Conakry, Conakry, Guinea; 7 Global Health Program, McMaster University, Hamilton, Canada; 8 National Research Ethics Committee of the Republic of Guinea, Conakry, Guinea; 9 Ministry of Health, Republic of Guinea and Université de Conakry, Conakry, Guinea; 10 Centre Médical & Conseil en Santé (CEMECO), Conakry, Guinea

## Abstract

With no cure and a high mortality rate, Ebola virus disease (EVD) outbreaks require preparedness for the provision of end-of-life palliative care. This qualitative study is part of a larger project on palliative care in humanitarian contexts. Its goal was to document and deepen understanding of experiences and expectations related to end-of-life palliative care for patients infected with Ebola virus disease (EVD) in West African Ebola treatment centres (ETCs) during the 2013–2016 epidemic. It consisted of 15 in-depth semi-structured interviews with individuals impacted by EVD in a Guinean ETC: either as patients in an ETC, healthcare providers, healthcare providers who were also EVD patients at one point, family relations who visited patients who died in an ETC, or providers of spiritual support to patients and family. Analysis was team based and applied an interpretive descriptive approach. Healthcare delivery in humanitarian emergencies must remain respectful of patient preferences but also local and contextual values and norms. Of key importance in the Guinean context is the culturally valued experience of “dying in honour”. This involves accompaniment to facilitate a peaceful death, the possibility of passing on final messages to family members, prayer, and particular practices to enact respect for the bodies of the deceased. Participants emphasized several challenges to such death in Ebola treatment centres (ETCs), as well as practices they deemed helpful to alleviating dying patients’ suffering. An overarching message in participants’ accounts was that ideally more would have been done for the dying in ETCs. Building on participants’ accounts, we outline a number of considerations for optimizing end-of-life palliative care during current and future public health emergencies, including for COVID-19.

## Introduction

The West Africa Ebola epidemic was the deadliest epidemic of Ebola virus disease (EVD) the world has ever seen, and one of the most significant international public health emergencies of 2014-2015. It started in December 2013 in the Forest region of Southeastern Guinea and subsequently spread to Sierra Leone and Liberia. It was categorized by a special committee of the World Health Organization as a public health emergency of international concern on August 8, 2014, and persisted until March 2016 when the end of the emergency period was finally declared (WHO, 2014).

Limited and chronically underfunded health facilities in affected countries were quickly overwhelmed by the influx of cases and a lack of protective equipment or ability to isolate patients, as well as few healthcare providers with past experience managing this highly infectious and fatal level four pathogen. Médecins Sans Frontières, the Red Cross, ALIMA, and the World Health Organization were some of the more prominent international humanitarian healthcare organizations that stepped up in Spring 2014 to support national efforts to contain a growing crisis. In terms of patient care, the international humanitarian healthcare response included the following: building and equipping Ebola treatment centres (ETCs); funding additional healthcare team positions for patient and ETC management; and mobilizing and bringing in non-national (expatriate) healthcare providers and technical support for the ETCs; as well as training local and expatriate ETC staff in context-specific infection control and patient treatment protocols designed for EVD as a level four pathogen.

The average mortality rate for the West Africa Ebola outbreak was 39.5% across the affected regions for the period of 2013–2016 (Schultz et al. 2016). Across the three main affected countries of Guinea, Liberia, and Sierra Leone, 28,652 infections and 11,325 deaths have been attributed to the virus (Schultz et al., 2016). As part of a global study of palliative care in humanitarian crises (Humanitarian Health Ethics, 2018), the authors conducted a qualitative study of end-of-life palliative care provision in Guinean ETCs. Of particular interest was the question of whether or not specific end-of-life care was provided by healthcare teams in ETCs, as well as how any end-of-life care provided was connected to local socio-culturally normative expectations for end-of-life care. While palliative care, including but by no means limited to end-of-life care, is increasingly acknowledged as core to humanitarian healthcare, there has been limited research on its integration within international humanitarian healthcare responses.

## Background

Progress has been made for the prevention and treatment of the Ebola virus disease since the West Africa epidemic (Dhama et al., 2018). Recommended treatment to those infected includes antivirals in 2020 as we write, even as supportive measures remain central to clinical management (Dhama et al., 2018; WHO, 2019; Lamontagne et al., 2018). Still, EVD is a level four pathogen: highly deadly, and highly infectious. As such, treatment of EVD patients requires their isolation to limit the spread of the disease, and the use of a head-to-toe personal protective equipment (PPE) (suit, eye and head protection, gloves, boots) by all personnel entering any patient assessment and treatment areas in the ETC. These conditions, combined with the context of a public health emergency represent particular challenges to patient care, especially where human resources are limited, patient needs are complex, patient loads are high, and scientific understandings of the virus are still emerging. Those challenges must be considered in planning for the care of patients at all stages of disease in ETCs, including end of life.

The West Africa EVD epidemic resulted in over 11,000 deaths, with a majority of these occurring in ETCs. While as low as 18% in high-income country settings, the average mortality rate from EVD during the West Africa epidemic was significantly higher: 39.5% (Schultz et al., 2016). The reality continues to be that many who become infected by EVD will die. This inevitability raises the question: how can healthcare teams support the dying, under the unique conditions of an isolation centre, such as an ETC, and in the midst of a public health emergency? The goal of this study was to document and clarify challenges, perceptions, and expectations related to end-of-life palliative care needs of patients in West African ETCs. Ultimately, we aimed to learn from those living end-of-life palliative care in ETCs first hand, as patients, providers, and/or relatives of patients who died, what was done well, and what more might be done to alleviate suffering for dying EVD patients in ETCs in this West Africa context.

Provision of palliative and end-of-life care in any public health emergency can be understood as a clinical but also moral responsibility. The world’s most widely accepted minimum standards for humanitarian response define “palliative and end-of-life care that relieves pain and suffering, maximises the comfort, dignity and quality of life of patients, and provides support for family members” (Sphere, 2018), as an essential component of good humanitarian healthcare planning and provision. Failure to plan for palliative needs in humanitarian emergencies opens up an unacceptable risk: that patients who are actively dying and unable to access or benefit from curative treatments will be left without care and experience increased suffering (Powell et al., 2017; Rosoff, 2015; Smith & Aloudat, 2017; Hunt et al. 2020). Yet, failure to learn about specific local needs and preferences for end-of-life care at both the patient and socio-cultural levels also poses a risk: that of assuming increased access and preparedness for end-of-life care provision in humanitarian crises implies the same elements of care, regardless of context.

The political, economic, and socio-cultural landscapes upon which the Ebola epidemic and response in Guinea were layered, presented additional challenges for the provision of palliative care in this context. Multiple and overlapping forms of structural violence have operated continually, albeit in changing ways, from colonization onwards in Guinea, as in Sierra Leone and Liberia (Leach 2015; Niang, 2014). Much attention has been dedicated to the material effects of this violence and include severe under-resourcing of affected countries’ health systems [30]. Chronic under-resourced healthcare facilities and limited trained, paid, and experienced healthcare personnel structurally impeded efforts towards provision of palliative care and indeed all care, as providers were forced to contend with a dearth of personnel, medication, equipment, and space, especially in the early days of the pandemic (Boozary, Farmer and Jha, 2014; Carrión Martín et al., 2016; Leach, 2015; Niang, 2014; Wilkinson and Leach, 2014; Wilkinson and Fairhead, 2016, Thiam et al., 2015). Deep inequality and poverty; personal experiences and community histories of social, economic, and political exclusion; and limited opportunities for people in affected communities to help shape the response contributed to fear and distrust in affected populations (Leach, 2015; Niang, 2014, Wilkinson & Fairhead, 2016). This fear and distrust were expressed in various ways, including through the sharing of ideas about potentially nefarious motives behind the interventions that were provided, and through avoidance of healthcare seeking in the face of symptoms (Carrión Martín et al., 2016).

Resistance to public health emergency response and messaging have been documented and tied to similar historical and ongoing experiences of exploitation and inequality in the 2016-2020 EVD outbreaks in the Kivu region of the Democratic Republic of Congo (Ilunga Kalenga et al., 2019; Richardson, 2019). The first-hand knowledge and reflection of end-of-life in ETCs in Guinea can be helpful in other settings that, while unique in some aspects, might face similar conditions of widespread distrust towards ETCs, national or international actors, and response strategies to infectious disease outbreaks.

Patient care during the West Africa Ebola response, as is the case in many humanitarian emergencies, was part of an international response. Non-national experts led the development of technical guidance for ETC set-up and patient management. A majority of ETCs were built, equipped, and staffed by international humanitarian healthcare organizations, so that healthcare teams included a combination of national and expatriate healthcare providers. The layering of expatriate resources and expertise onto national public health emergency responses does beg careful consideration of how responses to palliative end-of-life needs in specific international humanitarian emergencies attend to and integrate culturally specific expectations or preferences. While focused on EVD in one African country setting, findings have relevance to the care of patients in other high mortality, highly infectious disease outbreak settings, including COVID-19. COVID-19 is significantly distinct from EVD in its lower mortality, modes of transmission, symptom burden, and those most vulnerable once infected. Still, like EVD, COVID-19 can involve the patient’s rapid decline, higher than usual numbers of critically ill patients, and, if available, the use of PPE, specific architectures to limit infection, and isolation of patients in dedicated care units. Accounts of those who faced uncertain risks and realities of dying from Ebola in ETCs may harbour important lessons for those involved in delivering care to patients at risk of dying in the current pandemic.

## Data collection and sampling

This qualitative study applied an interpretive description approach (Thorne, 2016). It involved fifteen in-depth semi-structured interviews with individuals impacted by dying in a Guinean ETC: either as patients in an ETC (*N* = 2); national (*N* = 6) and expatriate (*N* = 1) healthcare providers; healthcare providers who were also EVD patients at one point (*N* = 3); family relations who visited patients who died in the ETC (*N* = 2); or providers of spiritual support to patients and family (*N* = 1). Participant details are represented in Table 1.

**Table 1:** Interview participants

#	Respondent role	Gender
1	Physician	M
2	Patient survivor/psycho-social worker	F
3	Physician/survivor	M
4	Patient survivor	F
5	Patient survivor	F
6	Physician	M
7	Nurse	F
8	Religious leader (Imam)	M
9	Family of deceased	M
10	Family of deceased	F
11	Nurse	F
12	Physician	F
13	Nurse	M
14	Patient survivor/physician	M
15	Physician (expatriate)	M

Qualitative methods are ideal for gaining detailed insight on lived experiences and perceptions of healthcare practices (Sandelowski, 2000). Semi-structured interviews are well suited to the study of healthcare practices that have yet to be documented in detail, as this interview format leaves room for participants to volunteer areas for discussion of which the researcher may not be aware. As noted earlier, our study was one of several sub-studies within a broader programme of research studying palliative care in humanitarian crises (disasters, refugee settings, public health emergencies). The interview guide was developed for and unique to the Guinea study, but does share with other sub-studies in this broader programme of research overarching goals of documenting and advancing understandings of current palliative care needs and practices in specific humanitarian healthcare settings.

Participants were recruited based on the following eligibility criteria: they had survived life-threatening illness in the ETC, were relatives of patients who had died in the ETC, and/or worked as healthcare personnel or providers in one or more ETCs during the EVD outbreak in Guinea between 2014 and 2016; and, they were willing to reflect on their experiences providing, surviving, or losing a relative in ETCs. Recruitment was conducted through the networks of the Guinean members of the research team. Individuals identified as having worked in a healthcare delivery capacity in one or more ETCs were invited via a telephone call to consider participation in the study, and an invitation was also extended to the Guinean Ebola Survivors Network for potential surviving patient participants, and relatives willing to speak to their experiences visiting family who eventually died in ETC. Recruitment and sampling were purposive, aiming for a balance of males and females, and a balance of healthcare providers (HCPs) and others with lived experience. Two healthcare providers were also survivors, including one survivor who entered the ETC as a non-healthcare professional and was subsequently hired to provide psychosocial support to other patients. Amongst healthcare professionals interviewed, we sought diversity in terms of the professional background of participants (physicians, nurses, or psycho-social support workers). Participants were recruited in or near the capital Conakry to feasibly allow for timely completion of in-person interviews. Recruitment occurred through two methods. For HCPs, SK obtained phone numbers of ETC employees in the capital region through the Guinean co-investigators’ healthcare professional networks. For non-healthcare professional recruitment, seeking to recruit survivors who had observed end-of-life palliative care, the study was advertised through the Guinean National Survivors’ Association. For both participant groups, SK called potential participants to explain the study. Several participants requested in-person meetings to further discuss the study and the implications of participation.

In-person interviews were strongly favoured over virtual interviews for two key contextual reasons: the study focused on death and dying—potentially fraught topics rarely discussed by healthcare providers in Guinea; and the interviews focused on Ebola—which remained a potentially difficult subject matter for many who lived the outbreak at the time of the interviews. Therefore, we deemed it important that the interviewer be able to “read” a participant’s body language and in this manner understand if and when a point in the conversation was becoming overly distressing to the participant. We also valued in-person interviews in order to enhance rapport and trust between the interviewer and participants. Rapport and trust are vital to the quality of an interview (Newman & Robertson, 2012). In our team’s experience, such a relationship risks not being established in a Guinean context if researchers do not present themselves in person. Finally, on a practical level, internet and phone connectivity can be unreliable in Guinea.

The interview guide for the Guinea study was developed by aa, bb, and cc [EN1] in dialogue with Guinean members of the research team familiar with conditions in Guinean ETCs (DD, EE, X[EN2]). The local research coordinator, SK, a socio-anthropologist with extensive semi-structured interview experience and fluent in local languages, led recruitment and conducted all but one of the interviews in French or Susu. One interview was conducted with an expatriate physician in English, and completed by the study lead, aa. Those agreeing to an interview signed a consent form prior to the start of the interview. Interviews lasted 21 to 140 min and were conducted between April and July 2018. Interviews were digitally recorded with participants’ permission. Sample size was initially set at 12, given the potential in qualitative analysis for rich detail and themes to emerge in even smaller samples (Thorne, 2016). An additional three interviews were added to this initial plan, when individuals originally contacted for interviews provided their agreement as data collection was ending.

## Analysis

Interviews were transcribed verbatim: the French and Susu ones into French by a transcriptionist in Guinea, and the single English interview in Canada. Transcripts of the French interview and the single English interview were verified for accuracy by a bilingual study coordinator in Canada. The single Susu interview’s transcription was verified for accuracy by the Guinean coordinator [SK]. One experienced bilingual researcher and the Canadian study lead [EN], two Canadian team members including an ethicist and experienced qualitative researcher [MH], and a palliative care physician [KB], as well as three Guinean researcher-clinicians [FBD, PD, OBS] and two Guinean health researchers [AAD, SK] contributed to the analysis, which involved interview transcript coding and interpretation. Two research team members [EN and SK] read through the transcripts several times independently, to identify preliminary themes. The first author [EN] then engaged in parallel coding of two unique transcripts with each of the following team members: PD, OBS, and AAD. Differences in the themes identified by pairs were discussed as a team in Guinea, and then again with Canada-based co-investigators [KB and MH], to reach a consensus on a preliminary codebook. EN led the subsequent analysis that was organized using the NVivo 11 software, consulting with Guinean and Canadian team members on emerging additional codes to inform the development of themes and sub-themes. The research coordinator in Canada [AC] performed an overall audit of the coding to verify and validate the fit of coded interview sections under specific themes and sub-themes. Six team members [EN, KB, and MH in Canada; and SK, PD, and FBD in Guinea] reviewed a summary of findings and reached a consensus on the most significant findings in the data.

## Limitations

This study involved a small sample, recruited through the Guinean National Ebola Survivors’ Network (RENASEG) and the healthcare provider network of the research team. At the time of data collection, Ebola remained a stigmatized and feared disease in the country. Those who agreed to an interview were willing to speak with a researcher about death and dying, Ebola, and the ETC. Patient/survivors recruited through the Ebola survivors’ network were individuals accustomed to sharing personal details of their Ebola experience: network members are regularly approached by researchers in the post-Ebola research economy. It is unclear how this might have oriented our sample and the perspectives shared. Further research with HCPs, surviving patients, and family perspectives would be helpful to deepen understanding of expectations and perceptions of end-of-life care needs and provision in Guinean ETCs. While participants spoke to experiences in ETCs in several cities and regions of the country, only one participant had worked in an ETC in the Forest region of Guinea. Given the most violent expressions of distrust of the EVD response were in this region far from the capital, and that is one of several culturally distinct areas in the country, it may be that perceptions and expectations of end-of-life care in ETCs in this region would be different. Finally, the perspectives of those who died in the ETC remain undocumented and are not represented in this study.

## Ethics

This study was approved by the National Health Research Ethics Committee of Guinea (CNERS No. 75) and the Hamilton Integrated Health Research Ethics Board (2017-1855).

## Findings

Participants’ accounts of end-of-life care in Guinean ETCs were harrowing. These narratives advance understanding of conditions, perceptions, and expectations of palliative end-of-life care in Guinean ETCs during the West Africa EVD outbreak along four main axes: (1) the emotional hardship of delivering care in a context of overwhelming loss of life; (2) challenges to the delivery of this care, stemming from the architectural, clinical, and social conditions of care delivery; (3) elements that did contribute, in HCPs’ eyes, in particular, to the alleviation of patients’ suffering; and (4) an ideal of “dying in honour”, sometimes achieved but also often unmet within ETCs. While the first three of these findings provide detailed insight on the conditions and specifics of palliative care in ETCs, the last finding, dying in honour, represents an overarching consideration for the provision of socio-culturally sensitive end-of-life care in the Guinean ETC context. A table (Table 2) capturing key issues discussed by participants can be found at the end of the article.

**Table 2:** Key issues discussed related to end-of-life palliative care for patients infected with EVD in Guinea

Leading issues discussed		Description
Main findings	Sub-findings	
The emotional hardship of delivering care in a context of overwhelming loss of life	Frequency and number of deaths	Increasing number of deaths was psychologically taxing for HCPs and survivors
	Sickness and death of close relationships	HCPs and patients witnessed deaths and suffering of close relations, contributing to feelings of fear and devastation
Challenges to the delivery of care, stemming from the architectural, clinical and social conditions of care delivery	Architecture of the ETC	ETCs mostly at or beyond capacity and physical structure of the ETC caused patients to be in close proximity to the dying and the deceased.
	Absence of disease modifying treatment	Outside of experimental trials, there was no available treatment—all care was supportive care aimed at treating symptoms to maximize chance of survival
	Prognostic uncertainty	Limited predictability of EVD infection outcomes, difficulty identifying patients likely to die
	Social isolation of patients in the ETC	Limited HCP and family contact with patients due to contact precautions and PPE
	Patient distrust and fear of HCPs	Some patients were convinced ETCs and HCPs were there to harm rather than help the patients leading to refusal of available care and additional suffering
Pain and symptom relief	Patient agitation distress	Patients’ agitation and inability to effectively address it could render administration of pain and symptom relief difficult
	HCP discomfort with opioids	HCPs seemed reluctant to use opioids even if available
	HCP training	Some HCP had limited experience with IV and opioid use
Patient friendship and kinship bonds	Psycho-social and practical support provided between patients	Patients described support provided by fellow patients in the form of solidarity, encouragement and help with personal care tasks as a source of comfort
Limited truth-telling	Possible? Therapeutic value of limited truth-telling	Limited truth-telling practised by some HCPs, patients towards fellow patients, and visiting relatives to preserve hope and, in participants’ interpretations, support recovery
An ideal of “dying in honour”, sometimes achieved but also often unmet with ETCs	Dying without family	Dying “alone” interpreted as without loved ones (i.e., family) was described with anxiety and a key characteristic of what made the risk of dying in ETC horrible in the eyes of participants
	Dying completely alone	In overwhelmed ETCs, some patients died with even no HCP at their side, a situation that was described as unacceptable
	Dying without customary post-mortem rituals	Some drew attention to the challenge in ETCs of enacting important customary rituals pre- and post-death, and the absence of such rituals as adding to family suffering

### The emotional and psychological (or psychosocial) hardship of delivering care in a context of overwhelming loss of life

The frequency and number of deaths profoundly impacted those on the front lines of the EVD outbreak in Guinea, and continued to haunt many at the time of the interview:

Life inside was so painful in that, we would be sent patients and they would die right before our eyes….Third day, fourth day, the Centre was almost full. But as they admitted people, people were also dying. (P2, patient/survivor and national HCP)

I still see the death before me, like that. I don’t even see you. I see myself in the centre, like that. I see myself with the deceased patients. I see myself with friends who passed. (Participant 6, national HCP)

Even with cholera cases we had had in Guinea, we did not have such elevated mortality rates. So, at the outset, with every death, we were crying. (Participant 12, national HCP)

The “all encompassing” nature of death in the ETC, as Participant 4, a patient/survivor, put it, was exacerbated by both stigma and pre-existing familial and workplace relationships with patients, which implied very real risks of death to themselves and loved ones.

EVD, deadly and posing an undeniable risk of contagion, was heavily stigmatized in Guinea as in other West African outbreaks throughout the epidemic.

It was my younger sisters and the children of my older sister that left for the ETC…I felt afraid with everyone talking about Ebola and in light of the stigmas surrounding the disease. Since I saw that this disease had reached my home, even implying my kin, I was thinking “we are all going to die.” That is why, at first I actually ran and hid. I was afraid that my siblings would die. (Participant 10, family of deceased)

On an interpersonal level, a number of participants had witnessed multiple family members, mentors, and colleagues falling severely ill and dying in quick succession. The collective burden of grief amidst the fear and chaos was simply overwhelming. At least one participant, a patient in the ETC, had witnessed death for the first time, and noted this experience rendered it all the more impossible to forget the dying:

Investigator: So there were many deaths?

Participant: Yes. We saw only that. You know when you are sick and you see others who are sick dying every day; you think it is easy to forget that? (Participant 5, patient/survivor)

The exceptionally high levels of mortality, fear, and personal risk that marked the EVD outbreak in Guinea constitute important dimensions to recognize as part of the unique conditions being navigated by healthcare providers caring for the sick and dying in Guinean ETCs. Even after the crisis abated, that collective grief and impact, both material and psychosocial, on numerous families, was devastating to a degree rarely experienced in other palliative illness scenarios. Bearing witness to the extreme interpersonal, community, and health system losses represented a deep and lasting burden on all involved.

### Challenges to the provision of satisfactory end-of-life palliative care in ETCs

Participants identified several constraints to the provision of good end-of-life palliative care in ETCs. These included the architecture of the ETC; difficulty of identifying patients likely to die (prognostic uncertainty); limited HCP and family contact with patients; and patient distrust and fear.

### Architecture of the ETC

From the moment I entered, I saw only death. My mind was focused entirely on death. Because when you enter even, it is the smell of death and suffering people. You see people screaming and lamenting their suffering, gravely ill without help. No one coming to see them. That is what frightened me… There were a lot of people in the room. Too many people. Too many cries. You know if someone is critically ill, those sounds hurt them.” (Participant 4, patient/survivor)

The people suffering were hitting the [plexiglass] windows, the tent, to be heard. (Participant 9, family of deceased)

At the height of the outbreak, from June to November 2014, ETCs in Guinea were for the most part at or beyond capacity. The number of seriously ill and dying were at record highs. Makeshift ETCs included space for dozens of patients in a room, sometimes lying on cots on the floor close together. Healthcare providers limited by PPE restrictions moved quickly between patients during their limited shifts in patient treatment areas of the ETCs. Many who died did so without a healthcare professional at their side. The sounds, smells, and sights under these conditions were horrific, in participants’ accounts.

The ETCs’ physical structure frequently meant patients were confronted with other patients, sometimes including members of their own families, dying alongside them. Some recalled with evident distress personnel preparing the bodies of the dead for removal from the Centre:

Especially at the beginning I thought of death. That worry starts in the waiting room, when everyone is waiting for the test results. You don’t know if it is positive or negative. If it is positive, how will you get out? You see in the corner the doctors taking the dead out in bags. You don’t know where they are taking them and you don’t know if you will be one of those cadavers. (Participant 4, patient/survivor)

The morgue was, like, over there. The centre was not so big as to allow for things to be well delineated. No. I had seen dead on TV, drawings I had seen, in the films, I had seen some. But not like that, so close. That had never been something I had experienced. No. No. No. No. (Participant 2, patient/survivor and national HCP)

It is really frightening to stay with the sick who one is talking to and then after they are dead and one stays with their body before the doctors come take them away. (Participant 5, patient/survivor)

The old man was hiccupping. Vomiting blood. When we came in, he screamed out “Hey! Hey! Mbara fago!” [I am dead] (Participant 13, national HCP)

Such witnessing compounded the burden of suffering experienced by patients, survivors, HCPs, and likely also those who died.

One participant noted that patients being able to observe the placement of the dead intact in body bags had one advantage. This dispelled rumors circulating at the time that organs of the dead were being harvested in ETCs for export to Europe. This is an interesting perspective, and important in the context of widespread rumors related to ETCs and the Ebola response more generally. Still, survivors’ accounts such as those above suggest proximity to the dying and the dead in the ETC was more than anything a source of distress.

### Prognostic uncertainty

One of the realities of EVD in 2013–2016 was the absence of any treatment (outside of experimental trials) available to treat the virus itself. As a result, all care was “supportive care” aimed at treating the negative effects of the virus on patients while they fought the disease to try to maximize their chance of survival (Lamontagne et al., 2018). Again and again, HCP participants emphasized that their goal was to “do everything possible” to help the sick survive. Palliative end-of-life care was not uppermost in staff’s mind: supporting survival was the priority of those HCPs with whom we spoke.

Palliative care is of great importance because, when well done, it can save lives. The mortality, the very high mortality rate associated with Ebola, can be reduced. (Participant 1, national HCP)

We refused to create an isolation centre [in the ETC, for those actively dying]. It was a consensus. The three physicians there categorically refused. We did not even discuss it. Everyone said ‘No, no, no. I cannot let my brother die. If I need to go with him, I even prefer to go with him. (Participant 12, national HCP)

We definitely fought. What we could do, we did. (Participant 11, national HCP)

A lack of care specifically geared to support end-of-life needs was further complicated by limited predictability of EVD infection outcomes. Highlighted by clinician respondents was the reality of prognostic uncertainty. Sometimes, even patients who were initially very ill did recover. Conversely, patients who initially seemed relatively well could deteriorate very quickly and die.

Prognostic uncertainty combined with the only treatment for EVD being supportive care meant no real distinction was made between care for the seriously ill and the dying in the ETC.

Unfortunately, several stories of untreated end-of-life pain were shared by participants. These cases resulted from an amalgam of factors including ETC clinical staff’s focus on trying to save as many lives as possible, limited human and material resources, and prognostic uncertainty. A lack of preparedness for palliative care provision does also seem to have played a role.

Participants did emphasize that when it was more certain patients were dying, attempts were made where possible to ensure they were not alone. These measures are discussed in greater detail below.

#### The challenge of end-of-life patient care under contact precautions and in PPE

As a whole, care provision to EVD patients is extremely resource intensive, but also requires the ETCs to have a very rigorous structure and approach to screening, isolation, treatment, and care of the dead to protect providers and the wider community. All of these factors force limits onto the care of the seriously ill, limiting HCP time and contact with patients in the ETC, and patients’ contact with their loved ones outside it.

Some patients arrived in the ETC with one or more members of their family. Others found some relief in recognizing a social connection in another patient. Many who entered the ETC, however, did so alone and found no one familiar inside. This social isolation of patients in the ETC poses a first crucial constraint on end-of-life palliative care, which would normally aim to facilitate the presence and participation of a patient’s close relations in their end-of-life.

You know, all those who are sick want to be treated for their sickness. That is why one goes to the hospital. But in the case of Ebola, it’s more than that. First, there is the solitude. You are not used to living sick when you fall ill. Members of the family line up to visit you [normally]. And in the ETC, it is different. No one comes, and no one touches anyone. I would want to see my parents, my children, every day by my side. But it was impossible, according to the doctors. That is what made me cry. (Participant 4, patient/survivor)

All the cases of death there, for me, what was even more, more shocking, was the case of the children. When you see a child there suffering, their mother cannot approach them, no one can come to them, that is pitiful. (Participant 7, national HCP)

All care, including palliative care, had to be provided while HCPs were in PPE. This meant that norms of rapid response were impossible.

We called the doctors to tell them to come. That there was someone already on the floor. On the floor after stumbling around. But, well, you know, the doctors, when you call for help, it takes at least 5 to 10 minutes for them to come in. Because there is the PPE. They need to put it on and carefully. But the time it took for them to come the guy was dead.” (Participant 2, patient/survivor and national HCP)

There were severe time constraints on how long HCPs had to complete tasks, demanding triage of care activities.

…You know, in a rich country setting, if you’re that severely ill you’re monitored constantly. Right. So even going in every two hours, even if there are five patients and they’re severely ill, it’s not much time spent at the patient’s bedside. (Participant 15, expatriate HCP)

Even if you want to stay, you leave. Because your goggles start fogging up…and you too are afraid of becoming contaminated. Your suit starts getting wet, you can continue. But when the goggles fog up, you start looking at getting out. (Participant 7, national HCP)

Clinical staff in the ETC work in pairs, so that when one individual in the pair has reached their limit, the pair departs. Limited staff also meant that care was often not possible at night, leaving long hours in between interactions with patients, and sometimes leaving patients to watch over the deceased while waiting for staff to come to retrieve the dead.

In the case of the doctor who died, he died at night. It was several hours before the other doctors come to take him, because they were not at his side. It is my fiancée who told them, “Someone who was screaming here is no longer speaking! Come see!” (Participant 9, family of deceased)

Many participants recalled with regret and grief cases of patients dying without any medical personnel in the room, and only other sick patients at their side.

### Distrust and fear of HCPs

Patients’ distrust and fear posed their own challenges to the provision of patient care. While not a barrier to end-of-life care only, many participants stressed the difficulty of alleviating symptoms in patients who were convinced ETCs and the HCPs within them were there to harm rather than help ordinary Guineans.

I remember when I was amongst the sick, there was a young man from [city] in the ETC who was sick, refusing everything. Panicked people everywhere [saying]: “Don’t eat! Don’t take the drugs! It’s poisoned.” There was a young man who also refused everything. He would be given drugs and he would throw them on the bed." (Participant 3, patient/survivor and national HCP)

There was the case of a woman who didn’t take her drugs. When the doctors would give her the drugs, she would take them in her hands and do as though she was taking them. Then, after the doctors would leave, she would dispose of them. I told her, “If you don’t take those capsules you will die.” She did not listen, and she died a few days after. (Participant 4, patient/survivor)

My family. They were saying, don’t drink any medicine they give you there otherwise you will die. If you consume the medicine of the whites, you will die. It is the white people and the doctors killing in the hospital. (Participant 5, patient/survivor)

There were others, that passed away, because they refused everything. They preferred to die than to accept anything. Even treatments. Even water to drink. Because for them, we were poisoning the water or the medication was poison. (Participant 12, national HCP)

### Factors contributing to the alleviation of patients’ suffering

Participants identified a number of factors that, though never eliminating the inordinately difficult conditions in the ETC, provided some relief to the seriously ill and dying. These included: administration of pain and other symptom relief, efforts to connect patients with their families outside the ETC, patients’ formation of friendship and fictive kinship bonds within the ETC, as well as concealing worsening patient conditions and deaths where these turns of events were judged to provoke too much further distress to patients.

### Pain and symptom relief

Very, very early, hurt or pain or distress was always at the center of our concern. (Participant 15, expatriate HCP)

Alleviation of physical and psychological suffering is central to a palliative approach. In the absence of curative pathways for EVD, one thing HCPs could do for patients was provide relief through drugs for pain, agitation, nausea, and diarrhoea. Still, the treatment of pain involved challenges. Some patients would have benefitted from IV fluids or medications but were too agitated or uncooperative due to confusion or absence of translators to discuss options or understand care plans. One expatriate HCP noted that some Guinean team members were reluctant to use opioids (injectable and intravenous) and IV lines more generally, possibly due to limited experience inserting these. This participant suggested reluctance to place IVs might also have been linked to HCP feeling needles with agitated patients presented too great a risk to their safety. The use of opioids was minimal, confirmed a Guinean HCP, who attributed this to “culture” (the statement was not probed by the interviewer). That HCP noted that while morphine was always available, it was only used once in their several months in the ETC with a pregnant woman in agony:

it was the only case. We had morphine but we did not use it. (Participant 12, national HCP)

Alongside treatment of physiological symptoms, several participants were explicit about the importance of continuing care and especially accompaniment to the dying.

When the indicators are so in the red, we do palliative care, and we need to accompany the patient up to their final breath…we have to maintain the mask of the human being. To keep, how can I say this in proper French? To humanize a person. Because even if I know they have to die, I need to accompany them. They need to die in all gentleness. That is what we call well planned palliative care. (Participant 6, national HCP)

Guinean healthcare professionals described accompaniment as a religious as well as clinical duty, in accordance with Islamic law interpreted as calling for treatment of the sick at all times:

When a sick person is down, they must be treated. No matter how slight the signs of life, they must be treated. Even Islamic law says a person must be treated, so long as they are breathing, eating, and talking. They must be treated. People with the means to do so must do it. Those with the knowledge must do it, to preserve the soul of that person. If God wants them to survive, good; but if they do not survive, also, you will have done what you could to help them. (Participant 8, religious leader)

Unfortunately, due to staffing shortages and limits on time HCPs could stay in PPE and with patients, supporting humane death through accompaniment to patients was not always possible. This weighed heavily on providers. Participant 15, an expatriate physician, spoke at some length, for example, about one case: a child, who had arrived at the ETC without parents or relatives and whose condition was deteriorating. They described surpassing the recommended time in the treatment area several times to be with this child and hold their hand, knowing the risk the child could die alone, and feeling this was unacceptable anywhere in the world. The child did eventually die without any provider or relative at their side: one case of a vivid loss and shortcoming of palliative care in the ETC participant 15 could not forget.

### Efforts to connect patients with their families outside the ETC

Visiting someone who is sick, that gives them hope. If you receive a family member at the ETC, for the rest of the day, your mind is at peace. It gives you the courage to heal quickly and finding yourself with members of the family. It was also like if we spoke on the phone. It gave me the impression I was already back in the neighborhood. (Participant 9, family of deceased)

Patients and HCP alike recognized the importance of connecting patients with families, as a key means of providing psychosocial support to patients and families. Phones were provided to patients with credit to telephone their family, on the understanding such a connection was indispensable to patient well-being and even potentially recovery. While prognostic uncertainty often rendered it impossible to predict which patients would die, HCPs did make efforts to alert any family members if one of theirs seemed close to death, to arrange a final visit. In these cases, the family member could see their loved one through the fence or visiting areas in some ETCs. The HCP also used such occasions to foreshadow to families an approaching likely death. In cases where deaths were more sudden, teams still contacted families by phone where possible, and again, if time allowed, inviting them to witness the preparation and removal of their loved one’s body after death. On such occasions, HCPs described their attempts to offer some words of comfort to the grieving, such as “We did everything we could for yours, but unfortunately, God did not want them to remain amongst us.” (Participant 7, national HCP).

Patients themselves sometimes seemed aware of their own imminent death, and requested their families be contacted. The following is one account of such a case:

It was the case of a well-known HCP…when he started taking a turn for the worse, we contacted his wife. His wife and children came at 7pm. He was sitting in the yard like this [facing the fence]. He had his eyes fixed on the road…It was as though he knew. He had his eyes fixed on the road. When his wife came she said: “Papa [husband], I am here. Papa, get well. We will leave this place.” His wife, his daughter said, “Papa, I am here.” It was his cherished daughter. Because he had asked me expressly – he had many children – but he asked that I get that daughter to come. She came. She said, “Papa, I am here. You asked for me?” He breathed in. He breathed out all at once. He was gone. He was dead. (Participant 12, national HCP)

Dying accompanied featured in all participants’ accounts of what set some deaths in the ETC apart as less distressing. It is a theme further expanded on in the section on dying in honour.

#### Patients’ formation of friendship and fictive kinship bonds within the ETC

One source of comfort in the ETC cited by numerous participants was friendship and a sense of family with fellow patients:

We were four survivors amongst the 19 who fell ill…we kept good relations there. We spoke with [name], and we spoke about the death of her son, her daughter, her husband. We discussed our household problems. We talked about everything…We talked to pass the time. You know, if you know someone in that situation, you are going to have a strong relationship forever. We became real friends and real family. (Participant 5, patient/survivor)

She would give me advice. She treated me like one of her sons. (Participant 3, patient/ survivor and national HCP, speaking of a fellow patient)

To limit infection, new arrivals at the ETC were prohibited to help one another while waiting for results: “Solidarity was forbidden,” as Participant 13, a national HCP, put it. Once determined positive, however, confirmed EVD patients became each other’s main source of support:

In the ETC, we were a family. We gave each other advice. About what? Oh, you, you are lying there, but you have to get up. You have to go for a little walk. Don’t worry about Ebola. You have to walk around. And I would visit some to see how they were doing. (Participant 14, patient/survivor and physician)

Survivors interviewed described various ways patients helped one another: to wash, to dress, to eat, to drink, walk around the centre, or accept medicine. Some cared for orphaned babies and, when strong enough, carried them on their backs as is the custom in Guinea. The nickname of “nounou” was given to recovering patients who stepped into roles of accompanying the dying in the absence of around-the-clock ETC staff presence. This nickname was also given to some survivors, seemingly immune to EVD post recovery, who were able to secure paid positions in the ETC to provide more general psychosocial support to fellow patients. Patients recognized in one another the commonality of their uniquely harrowing experience:

I know Ebola. I have had it. No doctor can tell me anything about that that I don’t know. Because as the saying goes, telling someone who has lost their mother: ‘I understand your pain’, you don’t. You have to lose your mother to understand. And I knew that pain. I had passed through it. (Participant 2, patient/survivor and national HCP)

### Limited truth-telling

Truth-telling related to prognosis, impending end-of-life, and death in the case of discussions with bereaved families, is a widely held principle in healthcare jurisdictions worldwide. In the Guinean ETC, it was not uncommon based on the accounts we heard to deviate from this practice. Limited truth-telling was practised by HCPs, fellow patients, and friends or relatives visiting from a safe distance. The principal reasons given for this norm in the ETCs was that this limited further distress to patients, and preserved hope deemed crucial to recovery in patients already struggling to battle the brutal virus.

You know, if your relative comes to the hospital because they are sick, it is good not to speak of frightening things that everyone knows already. (Participant 4, patient/survivor)

A doctor who had also been a patient in the ETC at one point, Participant 14, described the case of a young man who constantly asked after his mother and father who had entered the treatment centre with him. This patient was so worried for his parents, “especially his mother, because she had assured everything for this little one.” This participant, in his words, “did everything in the ETC so that [the young man] did not realize his parents had died” (Participant 14). When eventually a fellow clinician spoke the names of the dead in front of this young man/patient, Participant 14 was upset. For him, there “needed to be a strategy” to protect patients from such news. He told his younger colleague:

What you have done there, it’s serious. And it’s serious because, your words can really affect this young one. Your words can morally crush this young one. (Participant 14, patient/survivor and national HCP)

Relatedly, a couple of participants did not see added value in having priests or imams enter the ETC to supplement existing psychosocial supports. The presence of religious leaders was viewed by these individuals as something that would announce an impending death, and increase rather than alleviate patient distress:

Within religions, we know that when we ask the priest to intervene, it generally is towards the end. It is that there is no longer any choice, isn’t that correct?... And it wasn’t worth it actually. They [the patients] just needed hope: to believe [in possible recovery]. (Participant 2, patient/survivor and national HCP)

One female participant interviewed insisted no one told her she had Ebola explicitly, even after she was admitted to the ETC. She recalls simply being led into the zone where the confirmed cases were, and the healthcare staff telling her to take medications they offered her: “That is how I realized I was now someone sick with Ebola.” (Participant 4). Whether truth-telling depended on patients’ gender, and on the bases of what assumptions this might be, merits further research.

Seeking to protect patients may not have been the only reason underlying limited truth-telling. At least one HCP participant in our study could not explain why he had been unable to answer a seriously ill patient’s requests to be told truthfully their chances of survival:

He said to me, ‘tell me the truth.’ When someone asks someone who is sworn to tell the truth that, you realize what that means? Well, those words weighed on me every time I left [the room]. He was asking me to provide him with restitution: whether he would live or not. And I had the number. Eh!!! His numbers were very low. I knew he was going to die, but what day? I didn’t know. So, if he pushes me to tell the truth, you see? And so, I just stayed there tapping [taps his fingers together]. I couldn’t say it. I couldn’t find words to say it. (Participant 6, national HCP)

These are important findings to highlight, especially given that EVD outbreaks often do involve expatriate providers who may enter this context with differing norms and expectations. Such perspectives need to be examined in light of these findings to avoid unintended harms.

### Dying in honour

Many participants described the difference of end of life under ETC conditions of isolation, and in the face of the outbreak’s characteristic atmosphere of totalizing loss for those directly affected by the virus. Participants also, however, were asked explicitly in interviews: “How was death and dying different in the ETC, as compared to what might happen under normal circumstances in the Guinean cultural context?” and “What do you think should be done for individuals dying in the ETC?” A key theme or message from participants that emerged in response to these questions, and the interviews more generally was that a good death in Guinea involves “dying in honour”. Such dying involves, at its core, a re-iteration of the dying individual’s place in a social and human web of relations, and final affirmation of connection to their God. Intensely limited, but sometimes at least partially rendered possible through explicit efforts of ETC staff, “dying in honour” constitutes a key theme for thinking about how cultural norms may be supported under unique circumstances of isolation centres and a highly infectious disease outbreak.

Core to the possibility of “dying in honour” in participants’ narratives was non-abandonment.

We cannot do anything against death. We can approach, stay next to [the patient]. Then, administer the treatment, until death. But especially stay at their side. (Participant 11, national HCP)

The presence of loved ones at the bedside was described by several participants as core to Guinean notions of dying “ready” and in “honour”.

Imagine you die in Libya, there, out at sea. We retrieve your body, if found. Are we going to wash it? Maybe they cover it, but no, it’s not the same as if you were in your own family… It’s that to die in honour is to die surrounded by those who love you. To have the opportunity to ask them for forgiveness if you have wronged them. To accept their apologies if they have hurt you. To be able to say to yourself, yes, even if I must die, still I am ready. I feel close to my loved ones. (Participant 2, patient/survivor and national HCP)

In a home environment, family would often provide emotional and moral support, often in the form of prayers, but also personal hygiene and bedside assistance. Family could also massage the feet or body of the dying, “because the soul, it is difficult to remove the soul from the body.” (Participant 8, religious leader). The presence of family at the bedside would also typically, according to one participant, provide an opportunity for the dying to “put their life in order” (Participant 14, patient/survivor and local HCP) for example, asking forgiveness of some.

A number of participants stressed that most important in their view to the Guinean who is facing death is to be prayed over and to ask forgiveness: “It is what they need so that their soul can be removed in all gentleness.” (Participant 4). This is an opportunity to be granted forgiveness before death. It is also “so that their death can be calm because death is not easy,” as one provider put it. (Participant 9, family of deceased).

Beyond support and ritual, not being alone at the end of life, not being abandoned, also emerged as crucial to affirming the humanity of all involved: the humanity of the dying, of their loved ones, and of the providers who affirmed their commitment to humanity through a commitment to non-abandonment.

Investigator: But why is it important to be at the side of the dying?

Participant: “It is a human and they still have a soul within them.” (Participant 9, family of deceased)

What they must do for someone dying is they must watch over them, because it is a human being. God has respected the human being, and humans must also respect human beings. We must not abandon a dying patient to their family [in the ETC]. The doctors must let family know what is happening up until the sick person breathes their final breath. They must not abandon the sick to their family. (Participant 8, religious leader)

Dying alone was framed as tragic and morally unacceptable, but also in some accounts as existing on a continuum of dishonourable treatment with implications for the deceased even after death:

What is frightening, is to imagine that I am going to die and that maybe people will not even pray over my body. That, already …[trails off]. It’s…[trails off]. It is beyond the imaginable. I am going to die, and people will not even wash my body. I will not even have a right to a shroud? All of that, it is beyond what one can imagine. Because, we know that under ordinary circumstances when we die, at least we have, at the very least, we have those things. (Participant 2, patient/survivor and national HCP)

The thought of dying without customary post-mortem religious rituals was evidently deeply disturbing to some, and difficult for some to understand.

“It’s no good. Listen, everyone who died in that period was put in a bag. It’s not everyone who had Ebola! My sister said that the dead were not washed, and they were put in bags, regardless of how they died.” (Participant 10, family of deceased)

Below is one participant’s account of an “honourable” treatment of the deceased, in their neighbourhood and outside the ETC:

They treated him like a Muslim. They washed him well. You know, when he died, they sent him over to the morgue and washed him well before putting him in white dress as Muslims do. Leaving for the cemetery, he was placed in a pickup truck and the imams prayed over him. He was honored. (Participant 4, patient/survivor)

Participants stressed the importance of the deceased’s body not being exposed for all to see, in accordance with Muslim and local customs for the care of the dead.

Investigator: If you were in your final moments at the ETC, what would you want done for you?

Participant: I would want them to treat me well. That my body not be exposed to all as I saw for others. I would want them to respect my body as would be done at home. (Participant 5, patient/survivor)

Efforts were made to pray over the body in the ETC, sometimes with family present at a distance to witness these final rites. One HCP explained that, for them, practices such as not exposing the body for all to see and praying with the dying or for the deceased was more than respect for the patient and customs. It was also a matter of upholding rules whose compromise could bring consequences upon those who might ignore them: “We are sons of the country. If we don’t respect [those customs], it can act against us also.” (Participant 12, national HCP)

It should be noted that Guinea is culturally and ethnically extremely diverse. Key religions include Islam, Christianity, and indigenous religious beliefs there exist over two dozen ethnic groups with socially and culturally distinct practices and connections to parts of the land and the country’s history and current political landscape. Participants described “dying in honour” as a matter of importance to Guineans across these differences.

Finally, all but one of those interviewed stated that the nationality of the individual providing end-of-life care in the ETC mattered far less than the provider’s presence and perceived commitment to the patient. While derived from a small sample only, this seems important to highlight given limited understanding of whether or not the nationality of those providing for palliative care needs matters in the eyes of affected populations, but also because we know palliative care provision in ETCs during the West Africa outbreak was often provided by international teams, and in the context of many rumors linking Ebola and ETCs to foreign nefarious intentions, as noted in the background section of this paper. The one individual who did express a preference for national providers did describe deep gratitude to her expatriate care provider. She credits that person with keeping her psychosocially well enough to survive Ebola. Still, as she stated, the kinship of Guinean providers could be a comfort in her view: “As a citizen and when a compatriot is there, frankly, it is a sister who is there. That would please me more” (Participant 2, patient/survivor and national HCP).

## Discussion and conclusion

While the West African EVD epidemic was unique in many aspects, it also has important implications for palliative care in other public health emergencies and humanitarian crises, including for palliative care in the current COVID-19 pandemic. Similarities between EVD and COVID-19 should not be exaggerated. Significant differences between EVD and COVID-19 include but are not limited to modes of transmission, transmissibility, symptom burden, and mortality which directly impact the need for, approach to, and risks of palliative care provision. EVD affects low-resource settings and depends heavily on high-resource international humanitarian response for containment. The COVID-19 worldwide pandemic has impacted far greater numbers than EVD, while the response has relied mostly on highly inequitably resourced local health systems. Nonetheless, navigating rapidly changing patient scenarios and prognostic uncertainty has resulted in a similar need to provide active medical and palliative care simultaneously and seamlessly. Likewise, COVID-19 and EVD both involve navigating the architecture and particulars of infection prevention measures while maintaining vital human connections, including with family caregivers, for those facing the risk of imminent death. Participants’ accounts of what provided some comfort in the midst of what felt at times like chaos, and recommendations for what might be improved, are pertinent to planning and delivering care to patients at risk of dying in the current pandemic. The degree and specifics of this pertinence, however, will depend on context, as well as patient and family preferences.

The humanitarian imperatives to alleviate suffering and support human dignity were intensely present in the Guinean ETCs, but deeply challenged by contextual realities. The holistic principles of palliative care were recognized by all participants as essential to quality patient care within the ETC. At the same time, end of life palliative care in this setting was challenging due to considerations specific to a highly infectious pathogen whose evolution in patients was difficult to predict, a wider context of distrust towards the epidemic response amongst the affected population, generalized experiences of fear and loss amongst patients, as well as challenges stemming from limited human and material resources and the architectural set-up of ETCs.

The value of examining experiences from those on the front lines of the West African EVD epidemic lies in preparing for care to others in similar circumstances, while recognizing that global standards for palliative care in humanitarian crises can only go so far in defining best practice. Ultimately, local observations, understandings, and patient and family preferences, not global standards, will determine how care provided by humanitarian healthcare teams in a particular context is remembered and judged. Where the provision of humanitarian healthcare was seen to fall short by participants in this study, honest reflection can and should provide motivation and guidance to ensuring better care going forward.

Our findings support the assertion that palliative care must be integrated fully with treatment-focused, supportive care. While the value of early integration is encouraged and valuable in other illness trajectories, in EVD they are inseparable. Measures to address symptoms are inextricably intertwined with those intended to support survival (WHO, 2019; Lamontagne, 2018). This interconnection, coupled with prognostic uncertainty, means pragmatically that palliative care was being provided throughout patient care in ETCs in some form for almost all patients regardless of the ultimate outcome. This does not, however, obviate the need to attend to more intense and specific psychosocial and spiritual concerns of those who are dying, or more likely to die.

Attending to the specific elements that would facilitate more humane care in general will also extend to supporting better palliative care. The spatial architecture of ETCs was clearly a source of suffering in itself for patients and survivors. Measures wherever possible to limit proximity to extreme suffering and death of other patients, while maintaining functional and safe care environments for providers, are needed. Specific attention to how and where bodies were cared for was a specific source of distress. Modifications in protocols related to the management of the deceased were undertaken based on local guidance, and this is an important priority. Being unable to respond to alleviate suffering and death in a timely way is of course another aspect that extends beyond the spatial concerns. Staffing limitations in the ETCs were a central contributor to this reality. Measures to determine and meet requirements for the amount of staff and the infrastructure to support them need to be examined. Other strategies to optimize the use of PPE, timing of clinical encounters, and virtual communication between patients and providers would all serve to enhance the value of HCPs’ presence at the bedside. The value of their presence both psychologically and practically was a vital element to all care from our respondents’ experience.

HCPs themselves commented on their limited preparedness for the extent of palliative care needs, death, and dying they would be called on to address. The descriptions of patients palpably suffering without relief were and remain deeply disturbing. It is not clear to what extent various factors contributed in specific situations such as a lack of awareness and skills, availability of medications, staffing shortfalls and PPE limitations, cultural biases of providers and patients, policy, and procedural supports. However, the necessary components to an effective public health palliative care response include all of these (Stjernswärd, Foley and Ferris, 2007). The need for palliative care to be intentionally included in response planning and delivery is critical. This involves preparing for pain and symptom management, psychosocial aspects, and attention to cultural and patient-specific norms and preferences. Such planning needs to extend to staff training, drug and equipment availability, care policies and protocols, and ongoing clinical programme support. Such planning will not only enhance the quality of care being delivered but also support providers in mitigating their own moral distress and vicarious trauma (Hunt et al., 2018).

One notable adaptation in ETCs that supported care delivery under the staffing, PPE, and infection control limitations was the critical role played by fellow patients as essential caregivers. In many ways, they filled gaps left by both family and HCPs as best they could. In some instances, this created deep and lasting, almost familial, bonds that were essential to recovery in the ETC. We know from ongoing work in Guinea that ties established in the ETC between patients have also subsequently facilitated re-integration materially, psychologically, and socially for many survivors in the Guinean context. In addition, survivors, by virtue of their acquired immunity, were able to provide care more intimately and naturally than HCPs. They became key members of the team in providing psychosocial support for the dying and their loved ones and, importantly, a source of hope and trust to the sick. This unique role was a meaningful adaptation of care, and specific strategies to acknowledge and integrate it could be further enhanced.

The importance of the inclusion of local HCPs and leaders is one aspect of a wider finding highlighted through our interviews. While recognizing the need for a coordinated and international response, the intentional empowerment of local leadership in shaping the response can often be overlooked or undervalued. In the day-to-day care, our respondents spoke of the value of having Guinean providers alongside international providers, to enhance patient trust. The climate of mistrust, conspiracy, and fear was a significant contributor to breakdowns in patients seeking care, spread of the epidemic, and the ability to deliver care in ETCs. A failure to appreciate and address cultural and spiritual beliefs around illness, dying, and death can undermine public health responses at every level. Palliative care speaks into the crucial role of psychosocial and spiritual aspects of care being explicitly included and addressed wherever possible with open dialogue and efforts at mutual understanding and accommodation.

Specifically, this includes an honest examination of the cultural, ethical, and practical issues around disclosure. Our respondents called into question the dominant medical imperatives around disclosure of prognosis and even death of loved ones. They spoke of a strong, culturally accepted, imperative of compassion and protection. What is less clear is to what extent this may also be related to staff preparedness or experience for having these critical conversations.

In fact, underpinning these insights is a deeply held Guinean value placed on dying in honour. Humbly recognizing the inherent limitations of trying to explain a rich and unique cultural tradition, it encompasses key elements that can inform care beyond EVD and Guinea in important ways. Non-abandonment of the dying, for example, is an essential aspect relevant across humanitarian settings, as recent research has affirmed (Hunt et al. 2018). The presence with the dying wherever possible by family caregivers is also so crucial as an aspiration even in humanitarian crises. The ability of family and loved ones to provide truly holistic care embedded in lifelong relationships in ways HCPs cannot needs to be valued and supported to the full extent possible. Elements identified such as life review, reconciliation, forgiveness, and connection are deeply important in a once-in-a-lifetime passage. Clearly, infectious disease emergencies, and especially EVD, add complexity to actualizing this ideal. Nonetheless, accommodations and adaptions that prioritize maintaining connection were implemented and valued and should be enhanced wherever possible. Recent experience with COVID-19 has underlined the importance of balancing clinical and infection control priorities with consideration for patient and family psychosocial needs for one another (WHO, 2020; Pfefferbaum and North, 2020). Additionally, when it is truly not possible, the value of human presence from healthcare providers, or other patients as caregivers, are meaningful ways to continue to uphold the values at stake.

The vital importance of end-of-life spiritual rituals and care of the body is another aspect of Dying in Honour that needs careful attention. The value of these aspects to patients, families, and communities is often underappreciated in the medical maelstrom of public health emergencies. Still, modifications and adaptations to protocols should be made where possible to allow families and spiritual care providers in some capacity to meet these needs for those nearing death, and in the care of the body after death. Maintaining spiritual integrity must be balanced with personal safety. Adaptive approaches will require local wisdom and guidance to be given priority to be achieved successfully.

Taken together, the experiences of our respondents as patients, caregivers, family members, and healthcare providers provide important direction for the place of palliative care in ongoing response to Ebola and other infectious disease emergencies, and other humanitarian contexts. Table 3 provides a summary of recommendations issuing from this research, that we hope can support better preparedness for and delivery of palliative care in such diverse scenarios. The need to deliberately integrate palliative care capacity into humanitarian healthcare, to adapt responses to structural challenges, to prioritize local cultural understanding and leadership in structuring responses, and to give explicit value to psychosocial and spiritual aspects of care and to family caregivers as essential partners are highlighted.

**Table 3:** Recommendations drawn from findings

Recommendations	Description
Integrate palliative care with treatment focused, supportive care	In EVD, palliative and treatment-focused, supportive care are inseparable. These should be approached by HCPs as integrated aspects of all care
Ensure appropriate palliative medications and supplies are available	Plan for and aim to maintain adequate and appropriate medications and supplies to alleviate patient pain and suffering
Provide psychosocial supports to HCPs	Anticipate and ensure interventions/supports available to HCPs working in the high stress ETC environment
Enhance staff training and support where needed	HCP training in a number of areas is recommended: including training in palliative care/trauma informed care, opioid usage as part of a palliative approach, IV use, and critical/difficult communication
Consider the spatial architecture of ETCs	Maintain a functional and safe environment (ETC) while considering the proximity between patients so witnessing extreme suffering and death is minimized
Increase HCP numbers where possible	Increase HCP numbers for ETCs where possible, to increase time HCPs can be providing direct care, limit risks of patients dying alone, and alleviate pressure on individual HCPs
Prioritize hiring of local HCPs and their roles in guiding contextually appropriate care	Recognize that local HCPs familiar with the context of care delivery and local traditions are uniquely positioned to liase with patients and families to understand and help achieve care priorities and preferences
Plan for accompaniment of patients dying and at risk of dying	Accompaniment as death approaches is a moral imperative in many contexts, and, where absent, significantly increases suffering of patients, family, and HCPs. Given EVD’s high risk of mortality, ETC care delivery planning must include explicit strategies and sufficient staff to ensure the dying are accompanied
Explicitly discuss and determine harm vs. benefits of “truth” telling to patients	Recognize that in certain settings, some HCPs and family may wish to withhold information from a patient with the intention of reducing harm and improving survival chances for that individual. Where this is the case, rationales should be discussed openly in order to reach consensus where possible.
Facilitate wherever possible contact with patients and families	Provide cell phones loaded with credit to patients, should patients wish to communicate with family outside the ETC
	Plan time and resources for HCP provision of updates via phone to family of patients
	Where possible and the patient’s preference, encourage and help coordinate family visits, even if at a distance
Acknowledge and support fellow patients as caregivers	Actively engage and equip patients who are able in aspects of care provision during and after recovery.
